# Individual and combined association between nutritional trace metals and the risk of preterm birth in a recurrent pregnancy loss cohort

**DOI:** 10.3389/fnut.2023.1205748

**Published:** 2023-11-30

**Authors:** Yilin Liu, Tingting Wang, Yunpeng Ge, Hongfei Shen, Jiapo Li, Chong Qiao

**Affiliations:** ^1^Department of Obstetrics and Gynecology, Shengjing Hospital of China Medical University, Shenyang, China; ^2^Key Laboratory of Reproductive and Genetic Medicine, National Health Commission, Shenyang, China; ^3^Key Laboratory of Maternal-Fetal Medicine of Liaoning Province, Shenyang, China; ^4^Research Center of China Medical University Birth Cohort, China Medical University, Shenyang, China

**Keywords:** preterm birth, recurrent pregnancy loss, nutritional trace metals, Bayesian kernel machine regression, metal mixture

## Abstract

**Background:**

Recurrent pregnancy loss (RPL) was associated with an elevated risk of pregnancy complications, particularly preterm birth (PTB). However, the risk factors associated with PTB in RPL remained unclear. Emerging evidence indicated that maternal exposure to metals played a crucial role in the development of PTB. The objective of our study was to investigate the individual and combined associations of nutritional trace metals (NTMs) during pregnancy with PTB in RPL.

**Methods:**

Using data from a recurrent pregnancy loss cohort (*n* = 459), propensity score matching (1:3) was performed to control for covariates. Multiple logistic regression and multiple linear regression were employed to identify the individual effects, while elastic-net regularization (ENET) and Bayesian kernel machine regression (BKMR) were used to examine the combined effects on PTB in RPL.

**Results:**

The logistic regression model found that maternal exposure to copper (Cu) (quantile 4 [Q4] vs. quantile 1 [Q1], odds ratio [OR]: 0.21, 95% confidence interval [CI]: 0.05, 0.74) and zinc (Zn) (Q4 vs. Q1, OR: 0.19, 95%CI: 0.04, 0.77) was inversely associated with total PTB risk. We further constructed environmental risk scores (ERSs) using principal components and interaction terms derived from the ENET model to predict PTB accurately (*p* < 0.001). In the BKMR model, we confirmed that Cu was the most significant component (PIP = 0.85). When other metals were fixed at the 25^th^ and 50^th^ percentiles, Cu was inversely associated with PTB. In addition, we demonstrated the non-linear relationships of Zn with PTB and the potential interaction between Cu and other metals, including Zn, Ca, and Fe.

**Conclusion:**

In conclusion, our study highlighted the significance of maternal exposure to NTMs in RPL and its association with PTB risk. Cu and Zn were inversely associated with PTB risk, with Cu identified as a crucial factor. Potential interactions between Cu and other metals (Zn, Ca, and Fe) further contributed to the understanding of PTB etiology in RPL. These findings suggest opportunities for personalized care and preventive interventions to optimize maternal and infant health outcomes.

## 1 Introduction

Recurrent pregnancy loss (RPL), commonly defined as experiencing two or more consecutive failed clinical pregnancies, has emerged as a critical public health concern (1). Recent evidence suggest the association of RPL with an elevated incidence of adverse pregnancy outcomes, notably preterm birth (PTB) (2). Previous studies have reported that the occurrence of PTB in RPL ranged from 4.9% to 19.6%, significantly higher than the general population (3). However, the precise risk factors for PTB in RPL remained unclear. Growing evidence suggested that prenatal exposure to metals may exert a noteworthy influence on PTB (4, 5). Thus, in our study, we sought to explore whether exposure to nutritional trace metals (NTMs) also played a pivotal role in PTB among women with RPL.

A mounting body of evidence indicated the significant involvement of metal exposure in the development of PTB (6–13). Previous studies have primarily focused on investigating the individual effects of endocrine-disrupting or nutritional trace metals on the subsequent risk of PTB (14–18). However, adopting a “single metal” approach may not fully capture the complexity of interactions among metals. Instead of examining individual metal exposure, assessing the effects of multiple metal exposure, which offered a more comprehensive view of intricate interactions, non-linear associations, and combined effects, maybe more relevant and useful in predicting PTB (19).

Among previous studies that have examined maternal metal exposure in relation to PTB, only a few have evaluated the combined effects, and no study has investigated this association in RPL. Furthermore, these studies have yielded conflicting results. For instance, in a prospective cohort study, Wang et al. reported positive associations between magnesium (Mg), copper (Cu), and titanium (Ti) and PTB, while calcium (Ca), zinc (Zn), strontium (Sr), iron (Fe), and lead (Pb) showed negative associations (12). In a prospective birth cohort in rural Bangladesh, Huang et al. identified titanium (Ti), arsenic (As), and barium (Ba), which were detected in cord serum, as crucial predictors of PTB (7). Moreover, in the PROTECT cohort in Northern Puerto Rico, Ashrap et al. reported that lead (Pb), manganese (Mn), and zinc (Zn) detected in maternal blood were associated with an increased risk of PTB (6). However, Ren et al. indicated that Fe and Zn in hair had the strongest inverse effects on spontaneous PTB (13). In conclusion, the association between metal exposure and preterm birth varied with sample type, sampling time, composition of metal mixture, and statistical models.

Existing studies have mainly focused on the toxic effects of endocrine-disrupting metals. However, the effects of NTMs deserve attention, as they can be influenced by prenatal nutritional supplements and diet (20, 21). Additionally, maternal blood reflected long-term effects more accurately and was less susceptible to external impurities (22, 23). Other limitations from previous studies included the use of the single-metal model, which may not fully capture complicated interactions, non-linear associations, and combined effects (24). Finally, no studies have reported the association between NTMs and PTB in RPL.

To address these research gaps, our study aimed to investigate the association between NTMs in maternal blood and PTB in a RPL cohort in Northeast China. We hypothesized that there would be an inverse association between NTMs and the risk of PTB in RPL.

## 2 Materials and methods

### 2.1 Study population

We utilized data from the “Recurrent Pregnancy Loss Cohort Study (RPLCS)”, a sub-cohort of China Medical University Birth Cohort (CMUBC). The RPLCS was designed to examine the association between adverse exposure and mother–infant outcomes. In brief, we mainly recruited pregnant women with a history of RPL between May 2018 and January 2023 from Shengjing Hospital of China Medical University, which admitted the largest population of RPL patients in Northeastern China. We enrolled pregnant women who met the following inclusion criteria: women who were planning to conceive or had already conceived; had a history of pregnancy loss (≥2); without any mental disease; and agreed to participate in the project and follow-up. After the screening process, 1,588 women were deemed eligible to participate, out of which 947 agreed to participate. For the current analysis, we excluded non-RPL cases (*n* = 55), those with uterine anatomical abnormalities (*n* = 89), ineffective treatment of immune and endocrine abnormalities during pregnancy (*n* = 126), mother-paired loss to follow-up (*n* = 31), multiple pregnancies (*n* = 65), pregnancy termination (birth defects, abortions, or intrauterine deaths) (*n* = 116), and chronic diseases (*n* = 6). As a result, 459 singleton mother–infant pairs were included in the final analysis.

At the enrollment visit, face-to-face interviews were conducted using structured questionnaires administered by trained doctors to collect information on demographic and socioeconomic status and medical history. Subsequently, we collected the information from medical records and prospectively followed up until delivery, pregnancy termination, or loss of follow-up. Participants were followed up at 19.6 (±3.58) weeks of gestation for the detection of NTMs in maternal blood, including Cu, Zn, Fe, Mg, and Ca. All participants provided written informed consent, and the ethics committee of China Medical University approved the study.

### 2.2 Exposure: nutritional trace metals

Nutritional trace metals (Zn, Cu, Fe, Ca, and Mg) were detected in the second trimester (pregnancy weeks: 19.6 ± 3.58) in maternal blood. Sample collection, preservation, and detection were performed at the Central Laboratory of Shengjing Hospital of China Medical University. Detailed methods including: using atomic absorption spectrometry to measure the concentrations of Zn, Cu, and Fe and the methane-based xylenol blue (MXB) color development method and molybdate direct method to measure the concentrations of Ca and Mg separately (25). The NTMs were measured using the atomic absorption spectrometer system BH5300S (Boya (Beijing), China) and the ARCHITECT automated biochemical analysis system c16000 (Abbott, USA). The protocol was qualified by the China Metrology Accreditation (CMA) system. The units for Zn, Cu, and Fe were μmol/L, while the units for Ca and Mg were mmol/L. We converted the detection results to units of μg/L by multiplying with the molecular mass, enabling comparison with other studies. In the subsequent analysis, we transformed the continuous concentrations into quantiles.

### 2.3 Outcomes: preterm birth in RPL

The gestational week was calculated and corrected based on an ultrasound examination. In China, RPL was defined as two or more consecutive abortions (26). PTB was defined as a case group with a live birth within a gestational age between 28 and 36^+6^ weeks. Two subtypes of PTB were further distinguished: spontaneous preterm birth (sPTB) and iatrogenic preterm birth (iPTB) (27). Iatrogenic preterm birth refers to pregnancies that require early termination of pregnancy due to obstetric complications or medical comorbidities. Thus, we considered a successful pregnancy with PTB in RPL as the primary outcome. Conversely, women who delivered at ≥37 weeks in RPL were regarded as controls.

### 2.4 Covariates

The demographic and socioeconomic status and medical history of participants were collected at the time of recruitment. Pregnant women provided information about their age at enrollment, educational attainment, household income per month, and detailed adverse pregnancy histories through interviewer-administered questionnaires. We treated maternal age, pre-pregnancy BMI, spontaneous abortion frequency, and gestational weeks of detection as continuous variables. We categorized maternal educational attainment into three groups (senior high school or below, university or college, and postgraduate or above); household income per month (Chinese Yuan, ¥) into three groups (≤ 7,000, 7,000– <10,000, and >10,000); calcium supplement into four groups (600 mg qd, 600 mg bid, 600 mg tid, and unknown); iron supplement into two groups (yes vs. no); gender of infants into two groups (male vs. female); and hypertensive disorders in pregnancy (yes vs. no). We calculated pre-pregnancy body mass index (BMI) by dividing weight (kilograms) by the square of measured height (meters) and treated pre-pregnancy BMI as continuous variables (28).

### 2.5 Statistical analysis

To reduce heterogeneity in our study, we implemented propensity score matching (PSM) with a 1:3 protocol to minimize selection bias and control for potential covariates. Maternal age, gestational weeks of detection, pre-pregnancy BMI, education attainment, household income per month, calcium supplement usage, iron supplement usage, and number of abortions were used as matching factors. We adopted the nearest neighbor score matching principle and excluded four unmatched cases. Continuous variables were reported as mean ± standard deviation (SD), while categorical variables were reported as frequency (percentage). We compared PTB and control groups using the non-parametric test and the chi-square (χ^2^) test. Subsequently, we used the multiple logistic regression model to assess the association by calculating crude and adjusted odds ratios (ORs) and their 95% confidence intervals (CIs) for PTB and sPTB in RPL. Furthermore, the multiple linear regression model was also utilized to evaluate the association between concentrations of NTMs and gestational weeks of delivery.

To produce accurate estimates and avoid collinearity, we utilized elastic-net regularization (ENET) to identify significant metals as prediction markers (29). ENET combined the strengths of the Lasso and Ridge models, providing enhanced prediction power. For the ENET regression, we demonstrated the optimal values of α and λ through 10-fold cross-validation, aiming to minimize misclassification error. In this analysis, we only penalized the metal variables, while the covariates (same as the single-metal model) were included in the model. The NTMs with non-zero coefficients from the ENET model represented the key contributors driving the associations with PTB. Subsequently, we extracted the important components and interaction terms to calculate the environmental risk scores (ERSs) (30). We constructed the K–M survival analysis model to assess the association between ERSs and PTB in RPL, using PTB as the survival outcome and gestational week of delivery as the survival time.

Before establishing the BKMR model, we applied ln-transformation to the concentrations of NTMs (22). Bayesian kernel machine regression (BKMR) was utilized to estimate the overall effect, identify key components in the mixture driving the associations with PTB in RPL, capture the potential non-linear relationships, and identify interactions between the NTMs (31). The posterior inclusion probabilities (PIPs) can gauge the importance of each NTM. Variables with PIP values greater than 0.5 were considered statistically significant. We conducted component-wise variable selection for the five metals and evaluated the individual and combined effects with 50,000 iterations of the Markov chain Monte Carlo (MCMC) sampler (8). Furthermore, we validated the stability of our conclusions using the ROC curves. Additionally, we constructed the product of Zn and Cu concentrations and employed logistic regression to assess the combined effect of these two metals on PTB in RPL. All analyses were performed using R 4.2.2 software (R Core Team, Austria) and the following packages: MatchIt (version 4.5.0), glmnet (version 4.1.6), BKMR (version 0.2.2), and pROC (1.18.4).

## 3 Results

### 3.1 Characteristics of participants

**Figure 1:**
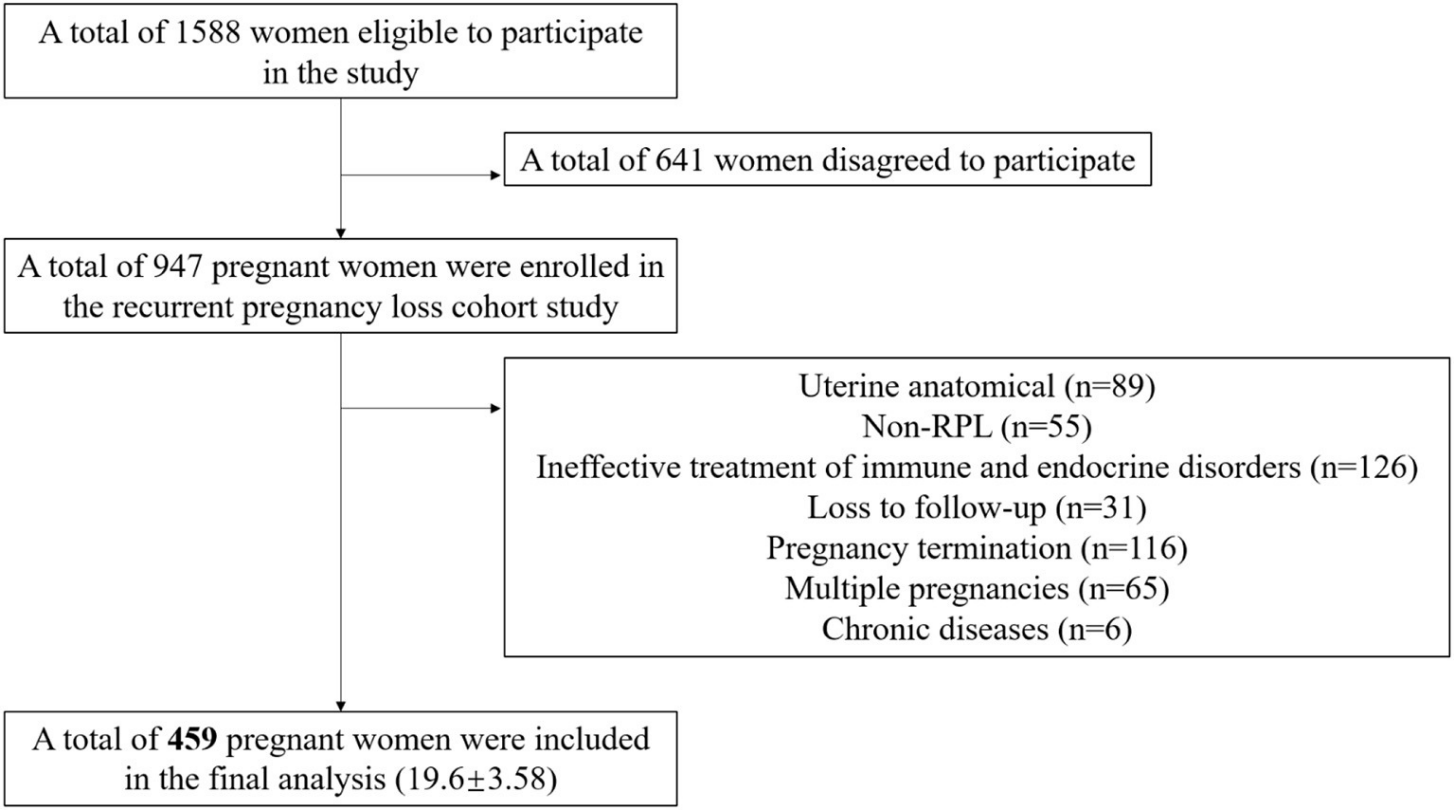
Flow chart.

In our study, 459 singleton mother–infant pairs were included in the final analysis (Figure 1). Moreover, 8.3% of participants experienced preterm birth (38 out of 459 participants). After applying propensity score matching (PSM) in a 1:3 ratio, some participants dropped out due to a lack of appropriate matching. At last, we included 94 controls and 34 PTB patients in the final analyses. Compared with term delivery, those with PTB had a slightly higher BMI (22.8 ± 3.65 vs. 23.9 ± 3.48) and were more likely to have lower levels of educational attainment (frequency of senior high school or below: 16.4% vs. 31.6%) and showed a lower likelihood of using iron supplements (11.4% vs. 0) in the total population. In the PTB group, participants tended to have more hypertensive disorders in pregnancy (5.9% vs. 18.4%). However, there were no significant differences in age at enrollment, household income per month, calcium supplement usage, or gestational weeks of detection between the two groups. We used PSM to minimize selection bias and avoid collinearity. In the PSM sets, no significant differences were observed between women with and without PTB in RPL (Table 1). The detailed concentration of NTMs in the included participants is presented in Supplementary Table S1. Additionally, we compared the concentrations of the NTMs with previous studies, as shown in Supplementary Table S2.

**Table 1:** Demographic characteristics of participants according to the case and control group in RPL.

**Variables**	**All participants**			**Propensity score-matched sets**		
	**Control (*****n*** = **421)**	**Preterm birth (*****n*** = **38)**	***p*** **value**^a^	**Control (*****n*** = **94)**	**Preterm birth (*****n*** = **34)**	***p*** **value**^a^
Age (years)	33.0 ± 4.8	32.9 ± 3.6	0.93	32.7 ± 3.75	33.5 ± 3.27	0.30
BMI (kg/m^2^)	22.8 ± 3.65	23.9 ± 3.48	0.07	22.9 ± 3.38	23.3 ± 3.33	0.55
Educational level (%)			0.07			0.91
Senior high school or below	69 (16.4)	12 (31.6)		22 (23.4)	7 (21.2)	
University or College	325 (77.2)	25 (65.8)		68 (72.3)	25 (75.8)	
Postgraduate or above	27 (6.4)	1 (2.6)		4 (4.3)	1 (3.0)	
Household income per month (CNY)			0.52			0.75
≤ 7,000	141 (32.8)	14 (36.8)		33 (35.1)	10 (30.3)	
7,000– < 10,000	208 (49.4)	20 (52.6)		47 (50.0)	19 (57.6)	
>10,000	77 (17.8)	4 (10.5)		14 (14.9)	4 (12.1)	
Calcium supplement usage (%)			0.99			NA
600 mg bid	231 (54.9)	14 (55.3)		150 (35.2)	14 (35.9)	
600 mg qd	146 (34.7)	21 (36.8)		232 (54.5)	21 (53.8)	
600 mg tid	42 (10.0)	4 (7.89)		42 (9.9)	4 (10.3)	
Unknown	2 (0.4)	0 (0.0)		2 (0.5)	0 (0)	
Iron supplement usage (%)			< 0.05^*^			NA
Yes	48 (11.4)	0 (0)		49 (11.5)	0 (0)	
No	373 (88.6)	38 (100)		377 (88.5)	39(100)	
Number of abortions	2.29 ± 0.77	2.53 ± 0.89	0.12	2.41 ± 0.932	2.48 ± 0.834	0.69
Gestational weeks of detection	19.6 ± 3.55	19.6 ± 3.63	0.80	19.4 ± 3.35	19.3 ± 3.78	0.85
Gestational hypertension			< 0.05^*^			0.77
Yes	25 (5.9)	7 (18.4)		12 (12.8)	5 (14.7)	
No	396 (94.1)	31 (81.6)		82 (87.2)	29 (85.3)	

a Nonparametric test and chi-square (χ^2^) test between the control and case groups.

BMI, body mass index.

* p < 0.05.

### 3.2 Single NTM and PTB in RPL: multiple logistic and linear regression models

Table 2 shows the associations between individual NTM and PTB risk in RPL, which are assessed using the multiple logistic regression model. The crude model (Model 1) was derived without controlling for covariates. After adjusting for potential covariates (Model 3), we observed that higher concentrations of Cu (quantile 4 [Q4] vs. Q1, odds ratios [OR]: 0.21, 95%CI: 0.05, 0.74; Q3 vs. Q1, OR: 0.23, 95%CI: 0.06, 0.79) and Zn (Q4 vs. Q1, OR: 0.19, 95%CI: 0.04, 0.77) were associated with a lower probability of PTB. However, no significant associations were found for other single exposures (Fe, Ca, and Mg). Similar results were obtained when associations between individual NTM exposure and sPTB in RPL were assessed. The concentration of Cu was associated with a lower risk of sPTB in RPL (Q4 vs. Q1, OR: 0.05, 95%CI: 0.002, 0.40; Q3 vs. Q1, OR: 0.22, 95%CI: 0.04, 0.9). However, consistent results were not observed for Zn in sPTB in RPL.

**Table 2:** The association between single nutritional trace metal and preterm birth.

		**Preterm birth (*****n*** = **34), OR (95%CI)**			**Spontaneous preterm birth (*****n*** = **19), OR (95%CI)**		
		**Model 1**	**Model 2**	**Model 3**	**Model 1**	**Model 2**	**Model 3**
Zn	Q1	1.00 (Ref)	1.00 (Ref)	1.00 (Ref)	1.00 (Ref)	1.00 (Ref)	1.00 (Ref)
	Q2	1.00 (0.34, 2.90)	0.97 (0.31, 2.95)	0.97 (0.32, 2.96)	1.50 (0.38, 6.57)	1.28 (0.30,5.87)	1.28 (0.30,5.93)
	Q3	1.15 (0.40, 3.32)	1.16 (0.37, 3.72)	1.13 (0.35, 3.64)	1.83 (0.48, 7.86)	1.33 (0.30, 6.39)	1.44 (0.31, 7.23)
	Q4	0.23 (0.05, 0.85)^*^	0.22 (0.04, 0.85)^*^	0.19 (0.04, 0.77)^*^	0.38 (0.05, 2.13)	0.31 (0.04, 1.82)	0.28 (0.02, 1.83)
Cu	Q1	1.00 (Ref)	1.00 (Ref)	1.00 (Ref)	1.00 (Ref)	1.00 (Ref)	1.00 (Ref)
	Q2	0.66 (0.23, 1.85)	0.60 (0.19, 1.82)	0.61 (0.19, 1.85)	0.48 (0.13, 1.64)	0.47 (0.11, 1.78)	0.43 (0.10, 1.63)
	Q3	0.34(0.10, 1.02)	0.25 (0.07, 0.84)^*^	0.23 (0.06, 0.79)^*^	0.32 (0.08, 1.16)	0.24 (0.05, 0.94)	0.22 (0.04, 0.90)^*^
	Q4	0.27 (0.08, 0.85)^*^	0.22 (0.05, 0.75)^*^	0.21 (0.05, 0.74)^*^	0.08 (0.00, 0.47)^*^	0.05 (0.00, 0.38)^*^	0.05 (0.002, 0.40)^*^
Fe	Q1	1.00 (Ref)	1.00 (Ref)	1.00 (Ref)	1.00 (Ref)	1.00 (Ref)	1.00 (Ref)
	Q2	0.38 (0.11, 1.23)	0.39 (0.10, 1.34)	0.35 (0.09, 1.24)	0.28 (0.04, 1.37)	0.23 (0.03, 1.27)	0.21 (0.03, 1.20)
	Q3	0.67 (0.22, 1.95)	0.65 (0.20, 2.08)	0.63 (0.19, 2.02)	0.61 (0.14, 2.42)	0.55 (0.12, 2.36)	0.53 (0.11, 2.37)
	Q4	0.91 (0.32, 2.58)	0.88 (0.27, 2.82)	0.85 (0.26, 2.78)	1.17 (0.34, 4.16)	1.06 (0.26, 4.40)	1.04 (0.24, 4.61)
Ca	Q1	1.00 (Ref)	1.00 (Ref)	1.00 (Ref)	1.00 (Ref)	1.00 (Ref)	1.00 (Ref)
	Q2	1.49 (0.49, 4.71)	1.55 (0.47, 5.34)	1.56 (0.47, 5.41)	1.25 (0.33, 4.86)	1.16 (0.27, 5.04)	1.20 (0.28, 5.17)
	Q3	1.00 (0.30, 3.33)	0.73 (0.20, 2.68)	0.74 (0.20, 2.75)	0.80 (0.18, 3.37)	0.59 (0.11, 2.82)	0.63 (0.12, 3.09)
	Q4	1.79 (0.58, 5.73)	1.62 (0.50, 5.49)	1.60 (0.49, 5.45)	1.00 (0.22, 4.27)	0.86 (0.18, 3.86)	0.84 (0.17, 3.83)
Mg	Q1	1.00 (Ref)	1.00 (Ref)	1.00 (Ref)	1.00 (Ref)	1.00 (Ref)	1.00 (Ref)
	Q2	0.84 (0.26, 2.67)	0.81 (0.24, 2.70)	0.84 (0.24, 2.85)	0.39 (0.05, 1.96)	0.38 (0.05, 2.08)	0.47 (0.06, 2.67)
	Q3	1.69 (0.54, 5.30)	1.57 (0.48, 5.25)	1.67 (0.50, 5.67)	1.20 (0.27, 5.14)	0.86 (0.17, 4.00)	1.07 (0.21, 5.26)
	Q4	1.61 (0.54, 4.90)	1.74 (0.55, 5.72)	1.73 (0.53, 5.70)	2.06 (0.60, 7.68)	1.84 (0.48, 7.49)	2.05 (0.53, 8.56)

Table analyzed using multiple logistic regression.

Model 1: crude model.

Model 2: adjusted for age, BMI, educational attainment, household income per month.

Model3: adjusted for Model 2 + number of abortions + hypertensive disorders in pregnancy.

OR, odds ratio; Q1, quantile 1; Q2, quantile 2; Q3, quantile 3; Q4, quantile 4; Zn, zinc; Cu, copper; Mg, magnesium; Ca, calcium; Fe, iron; BMI, body mass index.

^*^p < 0.05.

We further investigated the association between individual NTM and gestational weeks of delivery using the multiple linear regression model (Table 3). In the adjusted model (Model 3), the concentrations of Zn (β: 0.08, 95%CI: 0.06, 0.11, *p* < 0.05) and Cu (β: 0.32, 95%CI: 0.25, 0.38, *p* < 0.05) were positively associated with gestational weeks of delivery.

**Table 3:** The association between single nutritional trace metal and gestational weeks of delivery.

**Metal**	**Gestational weeks of delivery (weeks)**		
	**Model 1**	**Model 2**	**Model 3**
Zn	0.14 (0.12, 0.17)^*^	0.09 (0.07, 0.12)^*^	0.08 (0.06, 0.11)^*^
Cu	0.22 (0.16, 0.29)^*^	0.30 (0.23, 0.36)^*^	0.32 (0.25, 0.38)^*^
Fe	0.04 (−0.03, 0.10)	0.00 (−0.06, 0.07)	0.00 (−0.07, 0.06)
Ca	−0.01 (−1.69 1.67)	−0.02 (−1.76, 1.71)	−0.03 (−1.77, 1.72)
Mg	−0.06 (−1.84, 1.73)	−0.05 (−1.89, 1.78)	−0.05 (−1.90, 1.79)

Table analyzed using multiple linear regression.

Model 1: crude model.

Model 2: adjusted for age, BMI, education attainment, household income per month.

Model3: adjusted for Model 2 + number of abortions + hypertensive disorders in pregnancy.

OR, odds ratio; Q1, quantile 1; Q2, quantile 2; Q3, quantile 3; Q4, quantile 4; Zn, zinc; Cu, copper; Mg, magnesium; Ca, calcium; Fe, iron; BMI, body mass index.

### 3.3 Multiple NTMs and PTB in RPL: elastic-net regularization model (ENET)

**Figure 2:**
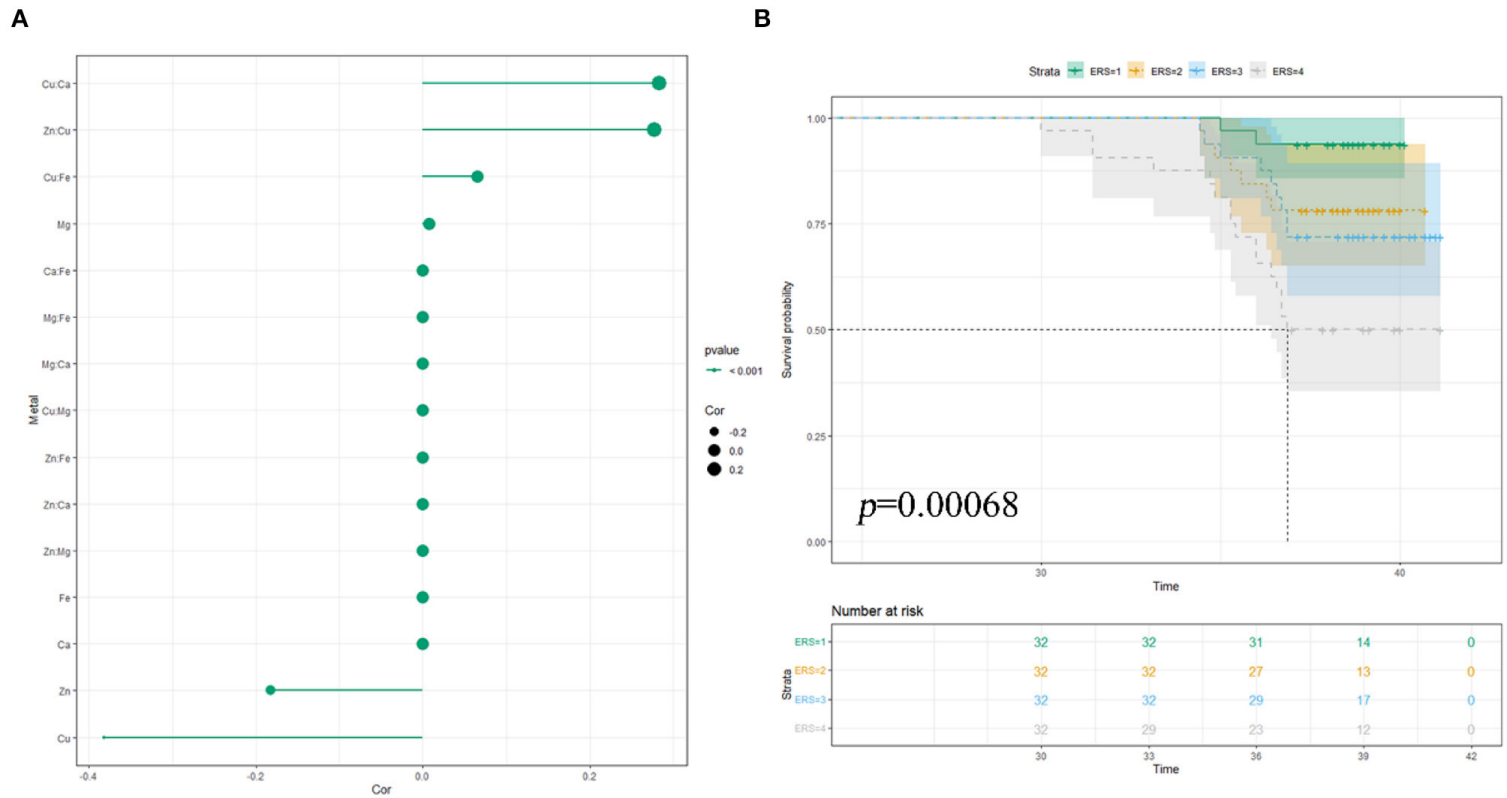
Multiple NTMs and preterm birth in RPL: elastic-net regularization model: **(A)** coefficient chart; **(B)** K–M survival analysis; model was adjusted for pre-pregnancy BMI, educational attainment, household income per month, age, number of abortions, and hypertensive disorders in pregnancy.

In the ENET model, we determined the main components and interaction terms using the ln-transformed and scaled values of the nutritional trace metals concentrations while adjusting for the same covariates in the single-metal model. λ and α were determined as 0.03795 and 0.9, respectively, through 10-fold cross-validation (Supplementary Figure S1). As shown in Figure 2A, the ENET model revealed non-zero coefficients (β≠0) for three individual components and three interaction terms after adjustment for covariates. Two metals (Zn and Cu) were inversely associated with PTB in RPL (β < 0), while Mg exhibited a positive association with PTB in RPL (β > 0). Notably, Cu showed the largest magnitude of β coefficient (β = −0.382), signifying the change in log-odds of PTB per increment in standardized ln-transformed and scaled metal concentrations. In addition, Cu demonstrated significant interactions with Ca (β = 0.283), Zn (β =0.277), and Fe (β = 0.066), all of which were positively associated with PTB in RPL. For further analyses, we constructed environmental risk scores (ERSs) according to the fitted ENET model and categorized individual ERSs into quartiles. As shown in Figure 2B, higher ERSs values were significantly associated with shorter gestational weeks of delivery in the K–M survival analysis.

### 3.4 Multiple NTMs and PTB: Bayesian kernel machine regression

**Figure 3:**
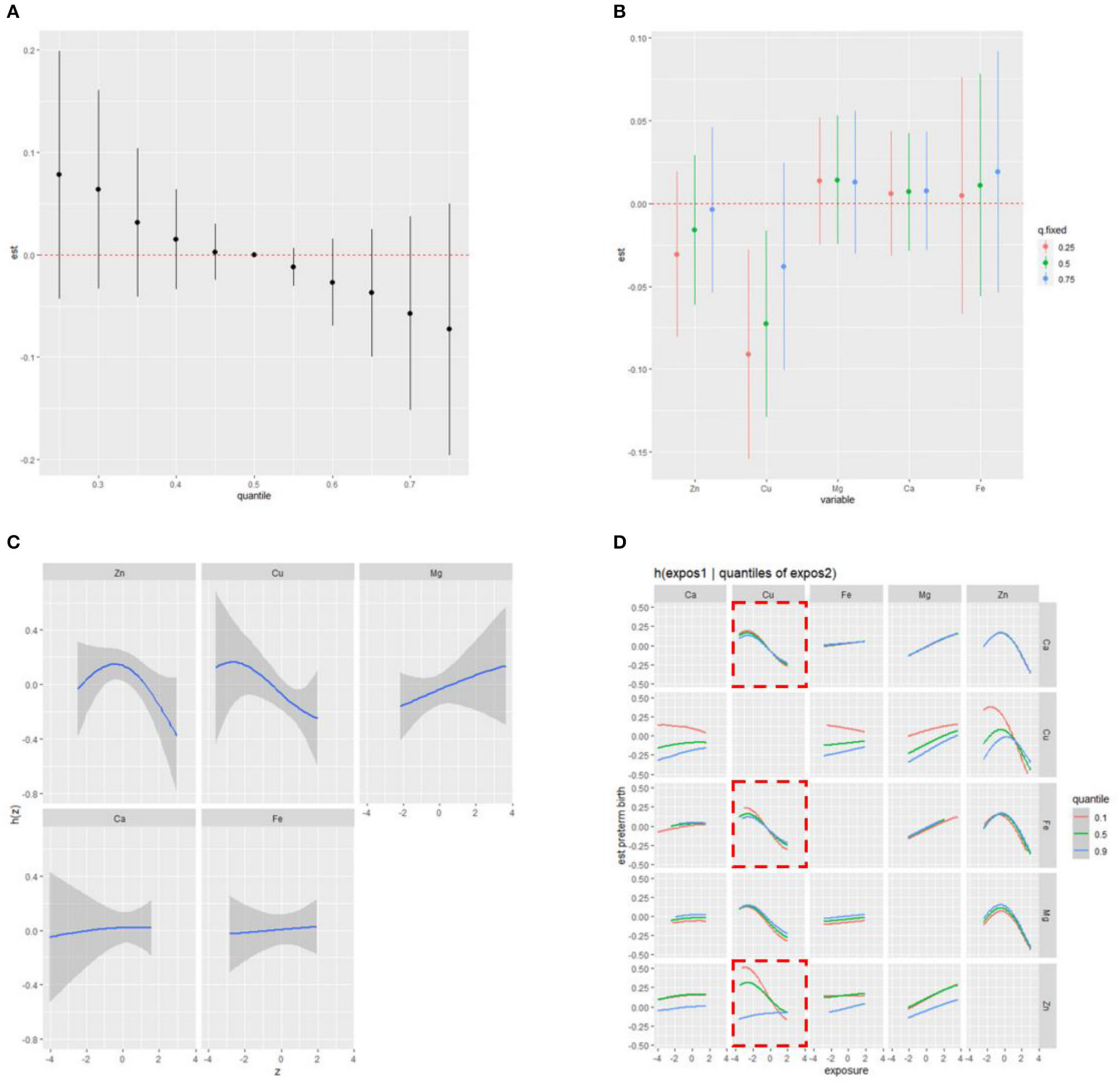
Combined effect of the NTMs on preterm birth in RPL estimated by BKMR **(A)** overall effect of NTMs on preterm birth (estimates and 95%CI); **(B)** independent effect of NTMs on preterm birth (estimates and 95%CI); **(C)** univariate cross-section (non-linear relationship); **(D)** bivariate exposure-response functions for each of two metals on preterm birth; model was adjusted for pre-pregnancy BMI, educational attainment, household income per month, age, number of abortions, and hypertensive disorders in pregnancy.

We first identified the significant metal components in the mixture and assessed their individual and combined effects on PTB in RPL in the BKMR model using the ln-transformed concentrations. Supplementary Table S3 shows that two metals, Cu (PIP = 0.84724) and Zn (PIP = 0.81200), were selected as significant variables in the mixture with PIP values >0.5. Figure 3A illustrates the combined effects of the metal mixture (comprised of five metals) on the latent continuous binary outcome of PTB in RPL. The results indicated a decreasing trend in PTB risk as the cumulative level across all metal exposures increased, although the findings were not statistically significant (*p* > 0.05). The independent effect of each metal on the mixed exposure is shown in Figure 3B. Visually, when the concentrations of other metals were constantly fixed at their 25^th^ and 50^th^ percentiles respectively, Cu was still significantly associated with a lower risk of PTB in RPL. Univariate dose–response relationships were estimated to explore potential non-linear correlations. As shown in Figure 3C, when each metal was fixed at its median value, Zn exhibited a non-linear association with PTB. Moreover, Zn had a negative linear relationship with preterm birth at higher levels (the confidence intervals at lower and higher distributions are wide due to sparse data). Additionally, Figure 3D demonstrates the bivariate exposure-response functions for each pair of metals on PTB in RPL. Notably, when Cu was fixed at the 25^th^ quantile, the slope between other metals (Zn, Fe, and Ca) and PTB was different from that when Cu was fixed at the 50^th^ or 75^th^ percentile, indicating the existence of potential interactions between Cu and other metals (Zn, Fe, and Ca). To validate the combined effect of Zn and Cu, we found that their interaction significantly reduced the risk of preterm birth (Q4 vs. Q1, OR: 0.14, 95%CI: 0.03, 0.54), as shown in Supplementary Figure S2. To validate the external applicability and robustness of our results, we conducted the ROC curve analysis (Supplementary Figure S3). The results indicated that total scores calculated by PIPs held predictive value in the overall population, particularly among mothers delivering female infants (AUC: 0.66, 95% CI:0.53, 0.79).

## 4 Discussion

In our study, we employed multiple logistic regression, multiple linear regression, elastic-net regularization model, and Bayesian kernel machine regression model to investigate the associations between maternal NTMs in the second trimester and PTB in RPL. Across all these methods, Cu was consistently identified as the most significant component among the NTMs and exhibited an inverse association with PTB in RPL. Furthermore, Cu showed a potential interaction with Ca, Zn, and Fe in the ENET and BMKR models. Moreover, the non-linear relationship between Zn and PTB was found in the BKMR model. Finally, we used K–M survival analysis and ROC curve analysis to assess the robustness of our results.

The study identified Cu as the most important factor among NTMs for PTB in RPL, which was similar to the previous findings in non-RPL. For instance, in a case–control study in Iran, Gohari et al. reported that Cu and Zn serum levels in mothers with preterm delivery were significantly lower than in mothers with term delivery (32). However, the sample collection was conducted at delivery, not in the second trimester. Moreover, Li et al. reported that lower Cu levels in the umbilical cord had a significantly higher risk of PTB (OR: 5.06, 95%CI:2.74, 9.34) and early-term birth (OR:1.36, 95%CI:1.10, 1.69) (33). No studies demonstrated the association between lower Cu levels in maternal blood in the second trimester and PTB in RPL. There were some inconsistent findings with our results. For example, Ashrap et al. confirmed no statistically significant association between maternal blood concentrations of Cu in the second trimester and PTB (6). Additionally, Hao et al. reported that serum Cu concentrations in the first trimester were positively associated with spontaneous PTB in a prospective cohort study in China (14). A recent meta-analysis showed that women with pregnancy loss showed significantly lower Cu concentrations than normal pregnant women, which demonstrated that Cu homeostasis was negatively associated with pregnancy loss (SMD = −1.42, 95% CI: −1.97 to −0.87, *p* < 0.001) (34). Furthermore, in the LIFECODES birth cohort, Kim et al. reported that maternal urinary Cu in the third trimester was associated with an increased risk of PTB (10). A possible reason might be attributed to differences in sample types and specific trimesters. Compared with our study in RPL [median (IQR): 1,131.92 (1,123.49, 1,520.66), μg/L], the investigations conducted by Ashrap et al. [mean (SD): 1,622 ± 1.25, μg/L] and Hao et al. [median (IQR): 1,720 (1,360, 1,980), μg/L] exhibited higher Cu (Cu) concentrations in their populations (6, 14). Conversely, Li et al. observed relatively lower Cu concentrations [median (IQR): 298.2 (123.1, 699.6), μg/L] in their study, akin to our findings (33). In other words, there may be a tendency toward an association between Cu and PTB. Low-dose exposure increases the risk of PTB; however, high-dose exposure may also elevate the risk of PTB. Previous studies indicated that increasing urinary Cu levels were associated with higher oxidative stress biomarkers (35). Excessive Cu excretion in urine led to insufficient antioxidant capacity, which might represent an important etiology for PTB. Recent studies have also shown that Cu deficiency is more prevalent in adult women than men in China (36). Therefore, the adverse effects of low Cu levels or even Cu deficiency on pregnancy outcomes deserved significant attention, especially for RPL.

Cu is an essential mineral that plays a crucial role in various physiological processes (37). The mechanisms underlying how lower levels of Cu contributed to PTB were multifaceted. Infection and aseptic inflammation were the main causes of PTB (38). Moreover, Cu was intricately involved in the development of immune cells and the signal transduction of immune responses. Thus, lower levels of Cu can lead to immune cell dysfunction and an increased risk of infection (39, 40). Furthermore, Cu played a significant role in neutralizing harmful free radicals and reducing oxidative stress (41, 42). As a component of enzyme structures, Cu was also essential in the synthesis of elastin and collagen, which contributed to enhancing uterine elasticity (43). Additionally, Cu was involved in the production of nitric oxide (NO) and angiogenesis, which helped regulate blood vessel dilation and blood flow to the uterus (44, 45). Interestingly, PTB and low-birth-weight infants tended to have lower Cu levels at birth, which may be related to maternal exposure to Cu deficiency. Understanding these mechanisms was important for developing effective strategies to prevent and manage PTB in RPL associated with Cu homeostasis (46).

Our findings unveiled a potential non-linear correlation between Zn concentrations and the risk of PTB in RPL. In a similar vein, Ashrap et al. demonstrated a comparable U-shaped pattern in Zn concentration, yet their findings indicated an association of higher concentrations with an increased risk of PTB (6). These non-linear patterns indicated that both excessive and insufficient exposure might have an impact on PTB, which underscored the intricacies of micronutrient interactions and their potential influence on pregnancy outcomes. Another important finding in our study was the potential interactions of Cu with Zn, Fe, and Ca, as revealed by both the ENET and BKMR models in RPL. This was consistent with the study by Liu et al., who reported similar potential interactions between Zn and Cu with PTB in non-RPL (8). The antagonistic relationship between Zn and Cu and PTB has been extensively studied. For example, Baecker et al. observed that Cu supplementation in pregnant female rats led to a significant decrease in brain Zn levels, particularly in the hippocampus (47). Furthermore, Kinnamon reported competition between Cu and Zn in the fetus and placenta, indicating the importance of optimizing the ratio of these elements during pregnancy to enhance reproductive outcomes (48). Interestingly, another case–control study conducted by Priya et al. demonstrated potential interactions between Fe and Cu in the case of polycystic ovary syndrome (49). Andersen et al. proposed that Cu deficiency not only affected the concentration of Fe but also indirectly impacted Fe transporters, which affected the delivery of Fe to the fetus (50). From the perspective of utilization, nutrient metals play a crucial role as signal transduction molecules in the development of the immune system, blood formation, antioxidant stress, cell differentiation, and apoptosis (51). They were involved in a complex regulatory network that controls these physiological processes.

There were several strengths of our study. First, we employed a population-based cohort design in the context of RPL, which allowed us to capture a representative and special sample in northeastern China, enhancing the generalizability of our findings. Second, to ensure the reliability and validity of our results, we employed multiple statistical models. Specifically, we utilized logistic regression, linear regression, the ENET model, and the BKMR model. Each model had its own unique advantages and limitations. This comprehensive approach strengthened the robustness of our conclusions and provided a more comprehensive understanding of the associations between NTMs and PTB in RPL.

Our study had several limitations. First, the sample size of our study was limited due to the difficulty of recruitment, which constrained the assessment of the association between NTM deficiency and PTB. Second, we did not apply the same detection method to various metals, which was distinguished from previous studies; however, it does not affect the accuracy of the results (8). Third, the concentration of NTMs was only detected in the second trimester, which may not reflect the concentration status of the first and third trimesters. However, limited studies investigated the exposure to the second trimester of PTB. Finally, there could still be some potential covariates due to unmeasured factors, such as maternal dietary information.

## 5 Conclusion

To summarize, we have recognized the individual and combined associations between NTMs and the risk of PTB in RPL. Detailed investigations revealed that maternal blood Cu and Zn levels in the second trimester were inversely associated with PTB in RPL. Additional studies are warranted to confirm these associations in RPL and understand the mechanisms behind the risk of NTMs and PTB in RPL.

## Data availability statement

Data presented in this study are available on request from the corresponding author.

## Ethics statement

The studies involving humans were approved by the Ethics Committee of China Medical University. The studies were conducted in accordance with the local legislation and institutional requirements. The participants provided their written informed consent to participate in this study.

## Author contributions

YL contributed to conceptualization, software, formal analysis, writing—original draft, investigation, supervision, project administration, and data curation. TW contributed to writing—original draft, writing—reviewing and editing, and project administration. YG contributed to conceptualization and writing—reviewing and editing. HS contributed to writing—reviewing and editing and project administration. JL contributed to data curation and investigation. CQ contributed to conceptualization, supervision, and funding acquisition. All authors have read and agreed to the published version of the manuscript.

## References

[1] Dimitriadis E Menkhorst E Saito S. Kutteh WH, Brosens JJ. Recurrent pregnancy loss. Nat. Rev. Dis. Primers. (2020) 6:98. 10.1038/s41572-020-00228-z 33303732

[2] Field K Murphy DJ. Perinatal outcomes in a subsequent pregnancy among women who have experienced recurrent miscarriage: a retrospective cohort study. Hum Reproduct. (Oxford, England). (2015) 30:1239–45. 10.1093/humrep/dev044 25759495

[3] Wu CQ Nichols K Carwana M Cormier N Maratta C. Preterm birth after recurrent pregnancy loss: a systematic review and meta-analysis. Fertil Steril. (2022) 117:811–9. 10.1016/j.fertnstert.2022.01.004 35131102

[4] Khanam R Kumar I Oladapo-Shittu O Twose C Islam AA Biswal SS. Prenatal environmental metal exposure and preterm birth: a scoping review. Int J Environ Res Public Health. (2021) 18:573. 10.3390/ijerph18020573 33445519 PMC7827269

[5] Singh L Anand M Singh S Taneja A. Environmental toxic metals in placenta and their effects on preterm delivery-current opinion. Drug Chem Toxicol. (2020) 43:531–8. 10.1080/01480545.2018.1515216 30257569

[6] Ashrap P Watkins DJ Mukherjee B Boss J Richards MJ Rosario Z. Maternal blood metal and metalloid concentrations in association with birth outcomes in Northern Puerto Rico. Environ Int. (2020) 138:105606. 10.1016/j.envint.2020.105606 32179314 PMC7198231

[7] Huang H Wei L Chen X Zhang R Su L Rahman M. Cord serum elementomics profiling of 56 elements depicts risk of preterm birth: Evidence from a prospective birth cohort in rural Bangladesh. Environ Int. (2021) 156:106731. 10.1016/j.envint.2021.106731 34197971 PMC11152765

[8] Liu J Ruan F Cao S Li Y Xu S Xia W. Associations between prenatal multiple metal exposure and preterm birth: Comparison of four statistical models. Chemosphere. (2022) 289:133015. 10.1016/j.chemosphere.2021.133015 34822868

[9] Wu Y Wang J Wei Y Chen J Kang L Long C. Maternal exposure to endocrine disrupting chemicals (EDCs) and preterm birth: a systematic review, meta-analysis, and meta-regression analysis. Environm Pollut (Barking, Essex: 1987). (2022) 292:118264. 10.1016/j.envpol.2021.118264 34606968

[10] Kim SS Meeker JD Carroll R Zhao S Mourgas MJ Richards MJ. Urinary trace metals individually and in mixtures in association with preterm birth. Environm Int. (2018) 121:582–90. 10.1016/j.envint.2018.09.052 30300816 PMC6233299

[11] Yu Y Gao M Wang X Guo Y Pang Y Yan H. Recommended acceptable levels of maternal serum typical toxic metals from the perspective of spontaneous preterm birth in Shanxi Province, China. Sci Total Environ. (2019) 686:599–605. 10.1016/j.scitotenv.2019.05.413 31185407

[12] Wang Z Huang S Zhang W Zeng X Chu C Li Q. Chemical element concentrations in cord whole blood and the risk of preterm birth for pregnant women in Guangdong, China. Ecotoxicol Environ Saf. (2022) 247:114228. 10.1016/j.ecoenv.2022.114228 36306619

[13] Ren M Zhao J Wang B An H Li Y Jia X. Associations between hair levels of trace elements and the risk of preterm birth among pregnant women: a prospective nested case-control study in Beijing Birth Cohort (BBC), China. Environ Int. (2022) 158:106965. 10.1016/j.envint.2021.106965 34735958

[14] Hao Y Pang Y Yan H Zhang Y Liu J Jin L. Association of maternal serum copper during early pregnancy with the risk of spontaneous preterm birth: A nested case-control study in China. Environ Int. (2019) 122:237–43. 10.1016/j.envint.2018.11.009 30473380

[15] Yang J Huo W Zhang B Zheng T Li Y Pan X. Maternal urinary cadmium concentrations in relation to preterm birth in the Healthy Baby Cohort Study in China. Environ Int. (2016) 94:300–6. 10.1016/j.envint.2016.06.003 27289180

[16] Li Z-J Liang C-M Xia X Huang K Yan S-Q Tao R-W. Association between maternal and umbilical cord serum cobalt concentration during pregnancy and the risk of preterm birth: the Ma'anshan birth cohort (MABC) study. Chemosphere. (2019) 218:487–92. 10.1016/j.chemosphere.2018.11.122 30497031

[17] Hu J Xia W Pan X Zheng T Zhang B Zhou A. Association of adverse birth outcomes with prenatal exposure to vanadium: a population-based cohort study. Lancet Planet Health. (2017) 1:e230–41. 10.1016/S2542-5196(17)30094-3 29851608

[18] Goto Y Mandai M Nakayama T Yamazaki S Nakayama SF Isobe T. Association of prenatal maternal blood lead levels with birth outcomes in the Japan Environment and Children's Study (JECS): a nationwide birth cohort study. Int J Epidemiol. (2021) 50:156–64. 10.1093/ije/dyaa162 33141187

[19] Braun JM Gennings C Hauser R Webster TF. What can epidemiological studies tell us about the impact of chemical mixtures on human health? Environ Health Perspect. (2016) 124:A6–9. 10.1289/ehp.1510569 26720830 PMC4710611

[20] Terrin G Berni Canani R Passariello A Messina F Conti MG Caoci S. Zinc supplementation reduces morbidity and mortality in very-low-birth-weight preterm neonates: a hospital-based randomized, placebo-controlled trial in an industrialized country. Am J Clin Nutr. (2013) 98:1468–74. 10.3945/ajcn.112.054478 24025633

[21] Zhang Y Xun P Chen C Lu L Shechter M Rosanoff A. Magnesium levels in relation to rates of preterm birth: a systematic review and meta-analysis of ecological, observational, and interventional studies. Nutr Rev. (2021) 79:188–99. 10.1093/nutrit/nuaa028 32483597 PMC8453627

[22] Ren M Yan L Pang Y Jia X Huang J Shen G. External interference from ambient air pollution on using hair metal(loid)s for biomarker-based exposure assessment. Environ Int. (2020) 137:105584. 10.1016/j.envint.2020.105584 32106049

[23] Ferguson KK McElrath TF Ko Y-A. Mukherjee B, Meeker JD. Variability in urinary phthalate metabolite levels across pregnancy and sensitive windows of exposure for the risk of preterm birth. Environm Int. (2014) 70:118–24. 10.1016/j.envint.2014.05.016 24934852 PMC4104181

[24] Lazarevic N Barnett AG Sly PD Knibbs LD. Statistical methodology in studies of prenatal exposure to mixtures of endocrine-disrupting chemicals: a review of existing approaches and new alternatives. Environm Health Persp. (2019) 127:26001. 10.1289/EHP2207 30720337 PMC6752940

[25] Costa FS Leal RVP Pacheco CSV Amorim FBAC de Jesus RM Santos LN. Multivariate optimization of an ultrasound-assisted extraction procedure for the determination of Cu, Fe, Mn, and Zn in plant samples by flame atomic absorption spectrometry. Analyt Meth. (2020) 12:2509–16. 10.1039/D0AY00554A 32930241

[26] Evaluation and treatment of recurrent pregnancy loss: a committee opinion. Fertil Steril. (2012) 98:1103–11. 10.1016/j.fertnstert.2012.06.048 22835448

[27] Goldenberg RL Culhane JF Iams JD Romero R. Epidemiology and causes of preterm birth. Lancet (London, England). (2008) 371:75–84. 10.1016/S0140-6736(08)60074-4 18177778 PMC7134569

[28] WHO Expert Consultation. Appropriate body-mass index for Asian populations and its implications for policy and intervention strategies. Lancet (London, England). (2004) 363:157–63. 10.1016/S0140-6736(03)15268-3 14726171

[29] Zou H HT. Regularization and variable selection via the elastic net. J Royal Stat Soc Seri B: Statist Methodol. (2005) 67:301–20. 10.1111/j.1467-9868.2005.00503.x

[30] Wang X Mukherjee B Park SK. Associations of cumulative exposure to heavy metal mixtures with obesity and its comorbidities among U.S. adults in NHANES 2003–2014. Environm Int. (2018) 121:683–94. 10.1016/j.envint.2018.09.035 30316184 PMC6268112

[31] Bobb JF Valeri L Claus Henn B Christiani DC Wright RO Mazumdar M. Bayesian kernel machine regression for estimating the health effects of multi-pollutant mixtures. Biostatistics. (2015) 16:493–508. 10.1093/biostatistics/kxu058 25532525 PMC5963470

[32] Gohari H Khajavian N Mahmoudian A Bilandi RR. Copper and zinc deficiency to the risk of preterm labor in pregnant women: a case-control study. BMC Preg Childbirth. (2023) 23:366. 10.1186/s12884-023-05625-2 37208743 PMC10197058

[33] Li Z Liang C Huang K Yan S Tao R Sheng J. Umbilical serum copper status and neonatal birth outcomes: a prospective cohort study. Biol Trace Elem Res. (2018) 183:200–8. 10.1007/s12011-017-1144-6 28856635

[34] Ren M Wang L Wen L Chen J Quan S Shi X. Association between female circulating heavy metal concentration and abortion: a systematic review and meta-analysis. Front Endocrinol (Lausanne). (2023) 14:1216507. 10.3389/fendo.2023.1216507 37711903 PMC10497972

[35] Domingo-Relloso A Grau-Perez M Galan-Chilet I Garrido-Martinez MJ Tormos C Navas-Acien A. Urinary metals and metal mixtures and oxidative stress biomarkers in an adult population from Spain: the Hortega study. Environ Int. (2019) 123:171–80. 10.1016/j.envint.2018.11.055 30529889

[36] Huang K Fang H Yu D Guo Q Xu X Ju L. Usual intake of micronutrients and prevalence of inadequate intake among chinese adults: data from CNHS 2015-2017. Nutrients. (2022) 14:4714. 10.3390/nu14224714 36432400 PMC9696081

[37] Uriu-Adams JY Scherr RE Lanoue L Keen CL. Influence of copper on early development: prenatal and postnatal considerations. BioFactors (Oxford, England). (2010) 36:136–52. 10.1002/biof.85 20232410

[38] Romero R Dey SK Fisher SJ. Preterm labor: one syndrome, many causes. Science (New York, NY). (2014) 345:760–5. 10.1126/science.1251816 PMC4191866 25124429

[39] Flatby HM Ravi A Damås JK Solligård E Rogne T. Circulating levels of micronutrients and risk of infections: a Mendelian randomization study. BMC Medicine. (2023) 21:84. 10.1186/s12916-023-02780-3 36882828 PMC9993583

[40] Gombart AF Pierre A Maggini S. A review of micronutrients and the immune system-working in harmony to reduce the risk of infection. Nutrients. (2020) 12:236. 10.3390/nu12010236 31963293 PMC7019735

[41] Hawk SN Lanoue L Keen CL Kwik-Uribe CL Rucker RB Uriu-Adams JY. Copper-deficient rat embryos are characterized by low superoxide dismutase activity and elevated superoxide anions. Biol Reprod. (2003) 68:896–903. 10.1095/biolreprod.102.009167 12604640

[42] Uriu-Adams JY Keen CL. Copper, oxidative stress, and human health. Mol Aspects Med. (2005) 26:268–98. 10.1016/j.mam.2005.07.015 16112185

[43] Dring JC Forma A Chilimoniuk Z Dobosz M Teresiński G Buszewicz G. Essentiality of trace elements in pregnancy, fertility, and gynecologic cancers-a state-of-the-art review. Nutrients. (2021) 14:185. 10.3390/nu14010185 35011060 PMC8746721

[44] Shukla N Thompson CS Angelini GD Mikhailidis DP Jeremy JY. Low micromolar concentrations of copper augment the impairment of endothelium-dependent relaxation of aortae from diabetic rabbits. Metabolism: Clini Exp. (2004) 53:1315–21. 10.1016/j.metabol.2004.05.007 15375788

[45] Wang Z Zhao Y Zhao Y Zhang Y Yao X Hang R. Exosomes secreted by macrophages upon copper ion stimulation can promote angiogenesis. Mater Sci Eng C Mater Biol. Appl. (2021) 123:111981. 10.1016/j.msec.2021.111981 33812609

[46] Wisniewska M Cremer M Wiehe L Becker N-P Rijntjes E Martitz J. Copper to zinc ratio as disease biomarker in neonates with early-onset congenital infections. Nutrients. (2017) 9:343. 10.3390/nu9040343 28358335 PMC5409682

[47] Baecker T Mangus K Pfaender S Chhabra R Boeckers TM Grabrucker AM. Loss of COMMD1 and copper overload disrupt zinc homeostasis and influence an autism-associated pathway at glutamatergic synapses. Biometals. (2014) 27:715–30. 10.1007/s10534-014-9764-1 25007851

[48] Kinnamon KE. Some independent and combined effects of copper, molybdenum, and zinc on the placental transfer of zinc-65 in the rat. J Nutr. (1963) 81:312–20. 10.1093/jn/81.4.312 14100990

[49] Sharma P Kapoor HS Kaur B Kamra P Khetarpal P. Investigation of the association of serum trace elements concentrations and serum biochemical parameters with the risk of polycystic ovary syndrome: a case-control study. Biol Trace Element Res. (2023). 10.1007/s12011-023-03664-6. [Epub ahead of print]. 37067720

[50] Andersen HS Gambling L Holtrop G McArdle HJ. Effect of dietary copper deficiency on iron metabolism in the pregnant rat. Br J Nutrit. (2007) 97:239–46. 10.1017/S0007114507239960 17298691

[51] Grzeszczak K Kwiatkowski S Kosik-Bogacka D. The role of Fe, Zn, and Cu in pregnancy. Biomolecules. (2020) 10:1176. 10.3390/biom10081176 32806787 PMC7463674

