# Supplementary material for: Individual and combined association between nutritional trace metals and the risk of preterm birth in a recurrent pregnancy loss cohort
Source: Front Nutr. 2023 Nov 30;10:1205748. doi: 10.3389/fnut.2023.1205748 (PMC10720726; doi:10.3389/fnut.2023.1205748)
Supplement: Supplementary file 1 [file Data_Sheet_1.docx]

Supplementary Material

Individual and combined association between nutritional trace metals and the risk of preterm birth in recurrent pregnancy loss cohort

Yilin Liu^1,2,3,4^, Tingting Wang^1,2,3,4^, Yunpeng Ge^1,2,3,4^, Hongfei Shen^1,2,3,4^, Jiapo Li^1,2,3,4^, Chong Qiao^1,2,3,4,*^

*** Correspondence:**Chong Qiao
[qiaochong2002@hotmail.com](mailto:qiaochong2002@hotmail.com)

**Table S1.** Distributions of nutritional trace metals (NTMs) in included participants.

| NTMs | Min | 25^th^ percentile | 50^th^ percentile | 75^th^ percentile | Max |
| --- | --- | --- | --- | --- | --- |
| Zn (μmol/L) | 59.03 | 78.01 | 83.72 | 95.92 | 135.8 |
| Zn (μg/L) | 3859.97 | 5101.07 | 5474.45 | 6272.21 | 8879.96 |
| Cu (μmol/L) | 7.56 | 17.68 | 20.96 | 23.93 | 34.50 |
| Cu (μg/L) | 480.41 | 1123.49 | 1331.92 | 1520.66 | 2192.34 |
| Fe (μmol/L) | 5.20 | 12.50 | 17.70 | 20.98 | 34.40 |
| Fe (μg/L) | 290.39 | 698.06 | 988.46 | 1171.63 | 1921.07 |
| Mg (mmol/L) | 0.60 | 0.80 | 0.86 | 0.92 | 1.67 |
| Mg (μg/L) | 14583 | 19444 | 20902.3 | 22360.6 | 40589.35 |
| Ca (mmol/L) | 1.47 | 2.17 | 2.24 | 2.32 | 2.59 |
| Ca (μg/L) | 58917.6 | 86817.36 | 89617.92 | 92818.56 | 103620.72 |

Abbreviation: copper (Cu), zinc (Zn), iron (Fe), magnesium (Mg), calcium (Ca), min(minimum), and max(maximum).

**Table S2**. Comparison of nutritional trace metal concentrations in related studies

| References | | Region | Outcomes | Sample | Period | Unit | Zn | Cu | Fe | Mg | Ca |
| --- | --- | --- | --- | --- | --- | --- | --- | --- | --- | --- | --- |
| Ashrap et al, 2020 (6) | Puerto Rico | | Preterm birth | Maternal blood | 2^nd^ trimester | μg/L | 4823±1.2 ^b^ | 1622±1.25 ^b^ | / | / | / |
| Huang et al, 2021 (7) | Bangladesh | | Preterm birth | Cord serum | Delivery | μg/L | 1209 ^a^  (1009, 1453) | 474 ^a^  (356, 681) | 3334 ^a^  (2441, 5236) | 21730 ^a^  (19669, 25185) | 103 ^a^  (96–112) |
| Liu et al,  2022 (8) | Wuhan, China | | Preterm birth | Urine | Before delivery | μg/g creatinine | 619.16 ^a^  (375.42, 980.20) | 20.21 ^a^  (13.27, 33.39) | / | / | / |
| Kim et al, 2018 (10) | USA | | Preterm birth | Urine | 2^nd^ trimester | ppb | 251 ^a^  (149, 379) | 9.22 ^a^  (7.08, 12.2) | / | / | / |
| Wang et al,  2022 (12) | Guangdong, China | | Preterm birth | Cord serum | 1^st^ Delivery | μg/L | 1477 ^a^  (1328, 1688) | 526 ^a^  (464, 597) | 459442 ^a^  (377635, 553392) | 43545 ^a^  (31661, 59099) | 32040 ^a^  (27332, 37326) |
| Ren et al, 2022 (13) | Beijing, China | | Preterm birth | Hair | 1^st^, 2^nd^, 3^rd^, 4^th^ trimester | μg/g | 242 ^a^  (193.1, 347.9) | 10.3 ^a^  (7.7, 14.26) | 22.5 ^a^  (17.9, 27.8) | / | / |
| Hao et al, 2019 (14) | Shanxi, China | | Spontaneous preterm birth | Maternal serum | 1^st^, 2^nd^  trimester | μg/dL | / | 172 ^a^  (136, 198) | / | / | / |
| Gohari et al,  2023 (32) | Iran | | Preterm birth | Maternal  serum | Delivery | μg/dL | 48.8±17.29 ^b^ | 166.90±62.27 ^b^ | / | / | / |
| Li et al.  2018 (33) | Anhui  China | | Preterm birth | Cord serum | Delivery | μg/L | / | 298.2 ^a^  (123.1, 699.6) | / | / | / |

Note:

^a^ Data were shown as median value (25% percentile -75% percentile);

^b^ Data were shown as mean value ± standard deviation;

**Table S3**. Posterior inclusion probability (PIP) estimated by BKMR model.

| NTMs | PIP^a^ |
| --- | --- |
| Cu | **0.84724**^b^ |
| Zn | **0.81200**^b^ |
| Fe | 0.28124 |
| Mg | 0.26720 |
| Ca | 0.24100 |

Note:

a. Posterior inclusion probabilities for each metal in component-wise variable selection;

b. PIPs were higher than 0.5;

c. Model: adjusted for age, BMI, educational attainment, household income per month, number of abortions, hypertensive disorders in pregnancy;

d. Abbreviation: nutritional trace metals (NTMs), posterior inclusion probability (PIP), copper (Cu), zinc (Zn), iron (Fe), magnesium (Mg), and calcium (Ca).


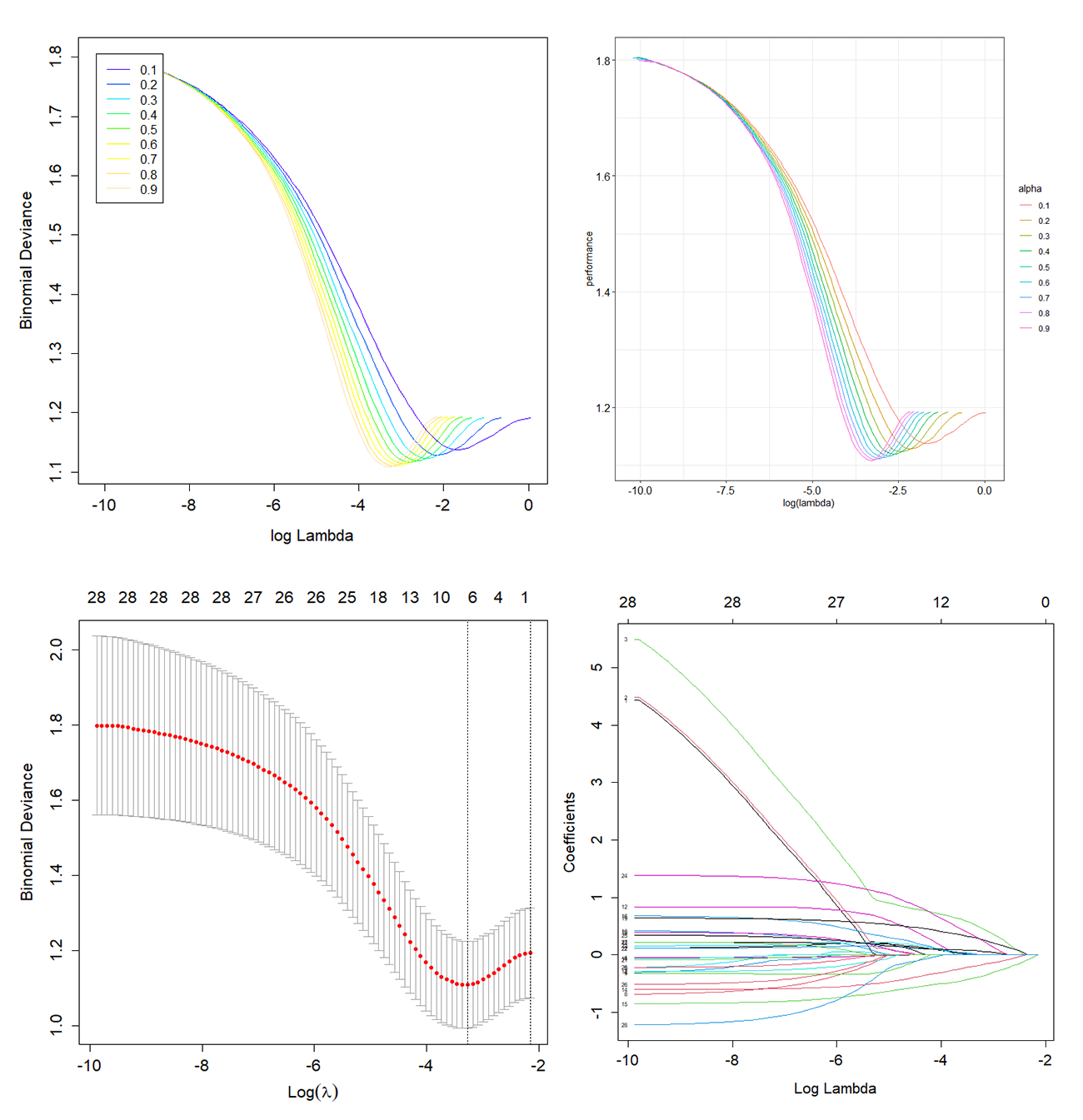


**Figure S1**. Construction of elastic net (ENET) regularization model: (A) The binomial deviance versus the log (Lambda) plots; (B) The performance versus the log (Lambda) plots; (C) Binomial Deviance of the ENET model with different log (Lambda); (D) ENET coefficient profiles.


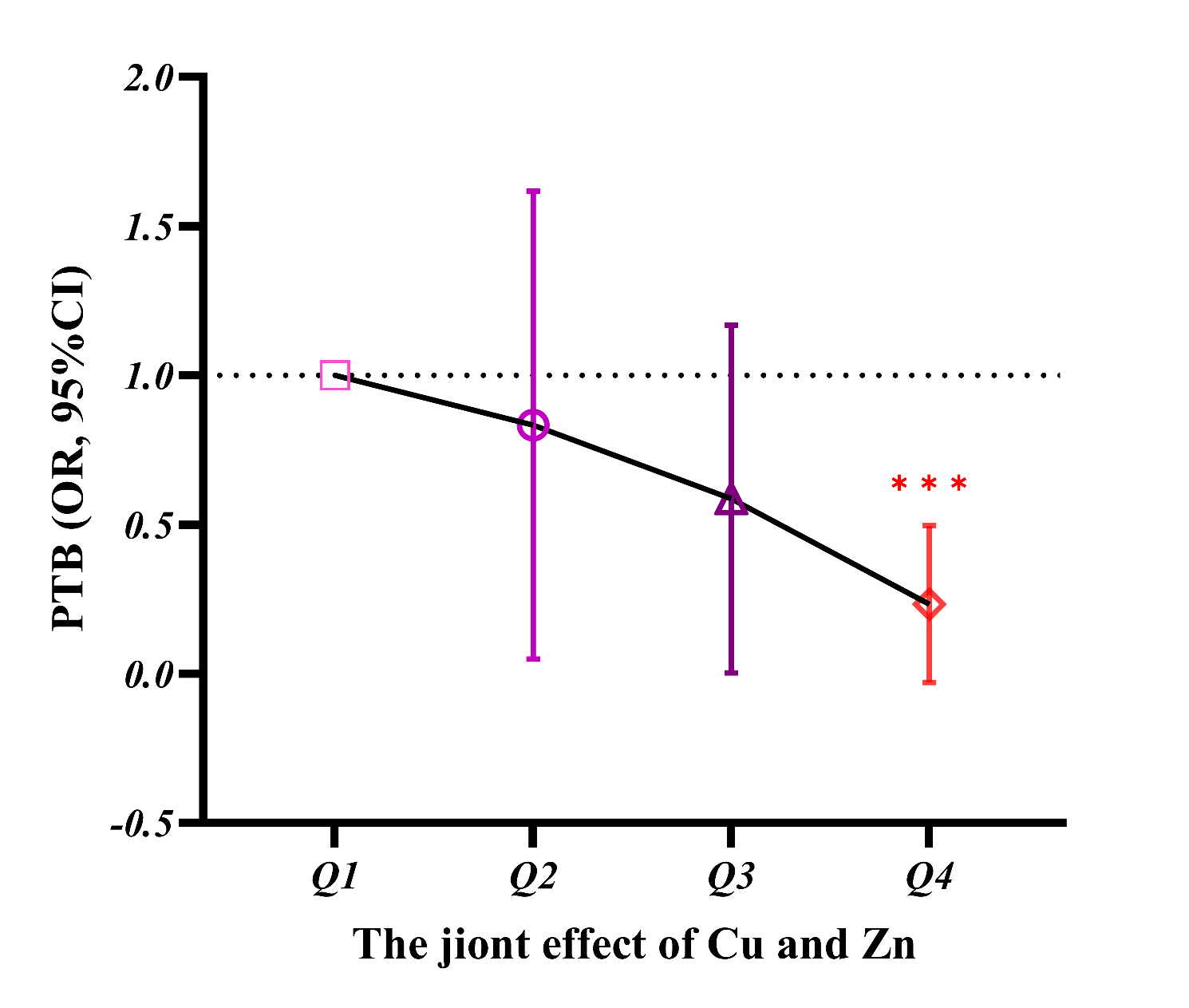


**Figure S2**. The joint effect between Zn & Cu and PTB in PRL.

Note: Figure S2 analyzed using multiple logistic regression.

Model: adjusted for age, BMI, educational attainment, household income per month, number of abortions, hypertensive disorders in pregnancy;

Abbreviation: OR: odds ratio, Q1: quantile 1, Q2: quantile 2, Q3: quantile 3, Q4: quantile 4, Zn: zinc; Cu: copper; ^*^ *p*<0.05, ^**^ *p*<0.01, ^***^ *p*<0.001.

**
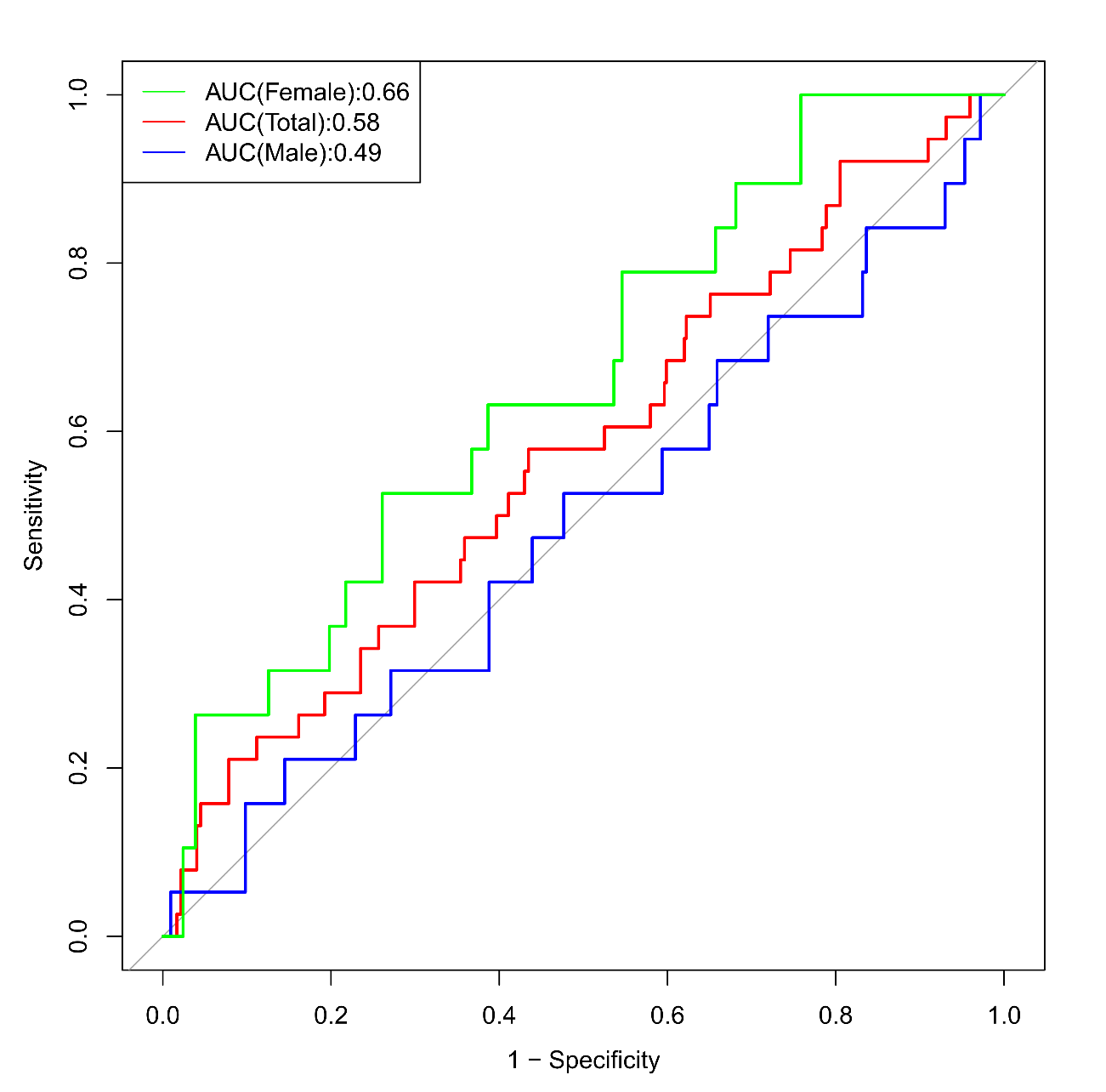
**

**Figure S3.** External validation in overall population (n=459)

$$Total scores=Cu\times0.84724+Zn\times0.812+ Fe\times0.28124+Mg\times0.2672+Ca\times0.241$$

1. The total scores were built based on the posterior inclusion probability (PIP) derived from Bayesian kernel machine regression (BKMR) model.
2. The continuous concentrations of all NTMs were log-transformed.
3. Stratified analysis: Female infants (green), Male infants (blue), and total population (red).
